# Axial separation simulation analysis and experiment of maize seed cylinder grading: A study on axial analysis

**DOI:** 10.1371/journal.pone.0337035

**Published:** 2025-11-21

**Authors:** Minji Liu, Jiannan Wang, Zhaoyan You, Chenyang Sun, Weiwen Luo, Ni Wang, Jianchun Yan, Hai Wei, Huijuan Zhang, Huanxiong Xie

**Affiliations:** 1 Nanjing Institute of Agricultural Mechanization, Ministry of Agriculture and Rural Affairs, Nanjing, Jiangsu, China; 2 Jiangsu Academy of Agricultural Sciences, Nanjing, Jiangsu, China; NED University of Engineering and Technology, PAKISTAN

## Abstract

This study conducted a simulation analysis of the seed grading process to address the challenges of excessive dependence on empirical parameter adjustments, low grading efficiency, and operational instability in maize cylinder grading systems. After determining the relevant characteristic parameters of maize seeds and the grading cylinder, discrete element models were established using EDEM software. The simulation investigated axial mean velocity, intra-cylinder quantity distribution, and post-sieving quantity distribution of seeds under varying cylinder speeds (14–22 r/min), inclination angles (0°–4°), and feeding rates (1,150–1,350 g/s). Orthogonal experiments and range analysis were conducted to determine the significance hierarchy of factors: for qualification rate, cylinder speed > inclination angle > feeding rate. For productivity, inclination angle > feeding rate > cylinder speed. The optimal parameter combination was cylinder speed 16 r/min, inclination angle 0°, and feeding rate 1,300 g/s. At this point, the qualification rate was 93.21%, and the productivity was 1,033 g/s. Using the optimal combination for validation experiments, the obtained qualification rate was 90.15%, and the productivity was 985 g/s. The relative errors between the experimental values and the predicted values were less than 5%, validating the simulation’s reliability and providing critical engineering implications for industrial seed grading optimization.

## Introduction

According to the data of the Food and Agriculture Organization of the United Nations (FAO), global maize production reached 1.248 billion tons in 2024, an increase of 8.7% over 2020. China’s maize production is about 290 million tons, accounting for about 24% of the global production, which plays a decisive role in ensuring national food security. Seed grading, a pivotal step in guaranteeing subsequent crop yields, employs various equipment configurations. Among these, the cylindrical grader is widely used in the grading operation of granular materials including maize seeds because of its simple structure, reliable operation and stable operation. However, the cylindrical grader also has the problems of low production efficiency and poor grading consistency, necessitating further optimization.

In response to these challenges, numerous scholars have conducted research on cylindrical grading systems. He [2024] proposed a variable-pitch cylindrical potato grading solution, which completed the design of core components including the grading cylinder, support wheels, and conveying device. Their study systematically analyzed the impact of different materials on the maximum falling height of potatoes, while investigating the influence patterns of various factors on grading quality to determine the optimal parameter combination [1]. Zong et al. [2019] examined cylinder sieve aperture size, inclination range, spiral blade structural parameters, and cylinder rotation speed range to explore the sieving characteristics of sunflower seeds in the cylinder. They analyzed each factor’s influence on operational quality and obtained optimal parameters through experiments [2]. Shi et al. [2018] improved a bar-type cylindrical nut grader, focusing on designing key components such as the transmission system, cylindrical sieving device, and blade adjustment mechanism, while calculating critical parameters including cylinder rotation speed, bar clearance, and lifting angle [3]. Yuan et al. [2006] combined theoretical analysis with experimental research to design a cylindrical nut grader and conducted tests to analyze the influence of different key parameters on operational quality [4]. Zhou [2012] developed a cylindrical jujube grader composed of multiple grading rings arranged side-by-side, utilizing the gaps between rings for grading. Through orthogonal experiments, they identified the main factors affecting performance and established a predictive model for grading quality [5]. Liu [2017] determined the structural and process parameters of a cylindrical sieve by analyzing material motion and sieving processes, and verified key transmission components using finite element analysis software. They conducted simulation tests with discrete element method software to optimize design parameters [6]. Hu et al. [2015] designed an adjustable-parameter cylindrical grader. After calculating the main structural parameters, they optimized operational parameters such as cylinder inclination, feeding rate, and rotation speed through orthogonal experiments, analyzing the influence of key parameters on tea leaf grading quality [7]. Li et al. [2019] created 3D geometric models of fresh tea leaves and a conical cylindrical grader using three-dimensional modeling software, established particle and contact mechanics models based on the discrete element method, and conducted numerical simulations with EDEM software to identify optimal technical parameters for high sieving efficiency [8].

However, existing research predominantly centers on optimization of cylindrical graders through parameter analysis, with a strong emphasis on equipment structural enhancements, the critical role of particle motion dynamics and spatial distribution patterns in determining grading performance has been largely overlooked—particularly for maize seeds. The cylindrical grading process for maize seeds constitutes a multifaceted interplay between particle mixing and separation mechanisms, whose fundamental principles remain poorly elucidated. Operational parameters exert their influence on grading outcomes primarily by altering the axial migration trajectories and distribution profiles of granular materials within the cylinder, yet systematic investigations into these phenomena are notably scarce. Moreover, the inherent working principle of cylindrical graders dictates that radial surface utilization is inherently constrained, while axial separation offers superior efficiency for seed grading. However, dedicated studies examining the axial movement and distribution characteristics of seeds during this process are extremely limited.

The Discrete Element Method (DEM) emerges as a uniquely powerful tool for addressing research gaps in maize seed grading, offering unparalleled capabilities in simulating multi-scale particle interactions with high fidelity. By enabling precise calibration of material properties, DEM provides a robust foundation for capturing the intricate nonlinear dynamics of axial particle transport, thereby facilitating real-time tracking of individual seed trajectories and uncovering microscopic movement patterns invisible in physical tests. This method not only reveals the direct correlation between seed migration patterns and grading outcomes but also enables high-efficiency multi-factor optimization through comprehensive parameter scans completed within 24 hours—a process that would require excessive seed consumption and days of labor in physical trials. Furthermore, DEM models ensure cost-effectiveness and reproducibility by eliminating material waste and manual sorting errors, as they require only initial calibration for infinite data reuse.

## Model establishment and parameter configuration

### Maize seed model

In order to construct the discrete element model of maize seeds, the ranges of the relevant model parameters obtained by referring to relevant literature [9] are shown in S1 Table.

**Fig 1:**
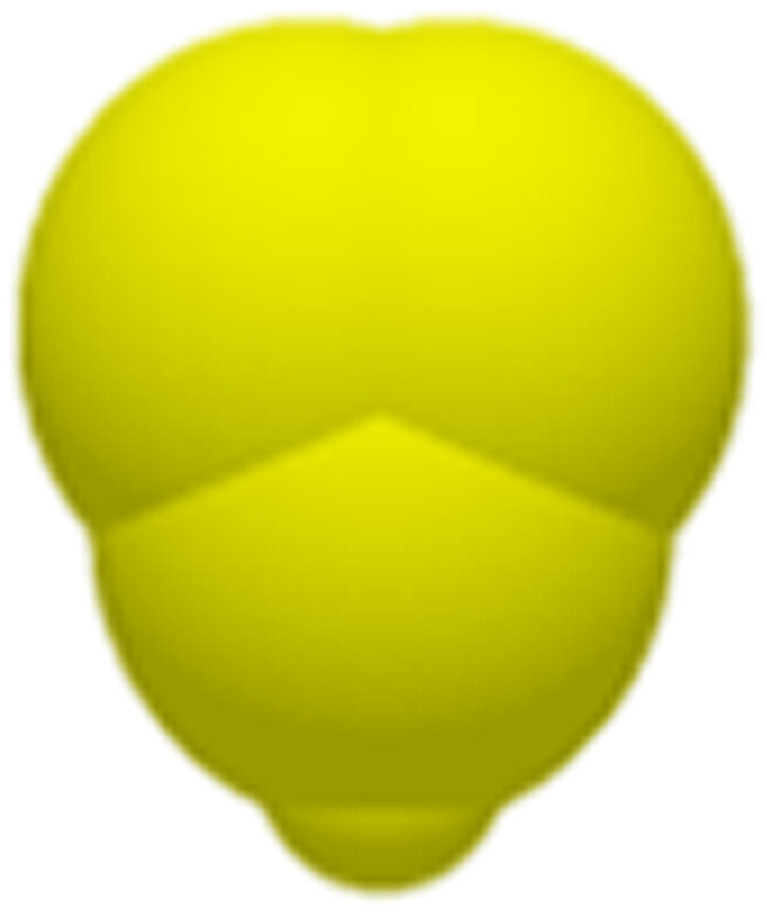
Simulation model.

**Fig 2:**
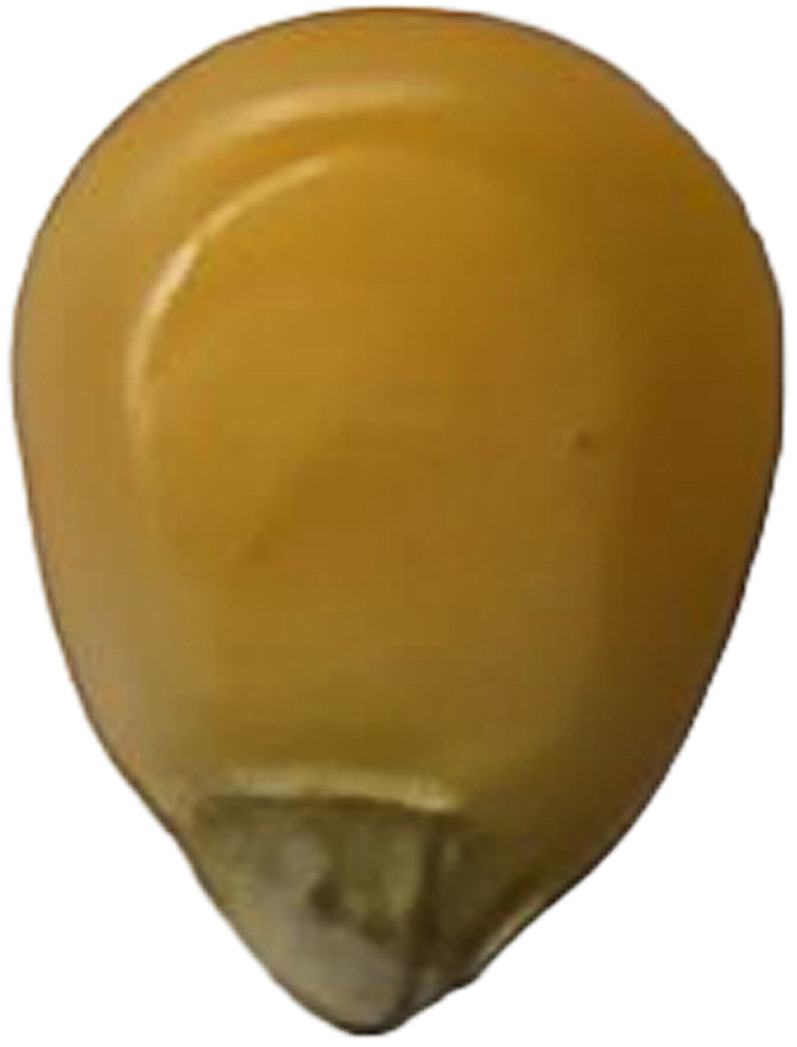
Maize seed.

Maize seeds for experiment were randomly selected and sieved using standard test sieves [10]. The sieved seeds were categorized into three groups based on width dimensions: large (width > 7 mm), medium (5 mm ≤ width ≤ 7 mm) and small (width < 5 mm). In EDEM 2018 software, the maize seed model was constructed using a sphere aggregation method [11,12]. Each seed model was consisted of four spherical particles. The simulation model and maize seed are shown in Figs 1 and 2.

### Grading cylinder model

The grading cylinder was designed with a 1,250 mm diameter and 3,000 mm length, featuring 7 mm diameter sieve holes. The cylinder material was specified as mild carbon steel. The relevant model parameters obtained by referring to relevant literature [9] are shown in S2 Table.

**Fig 3:**
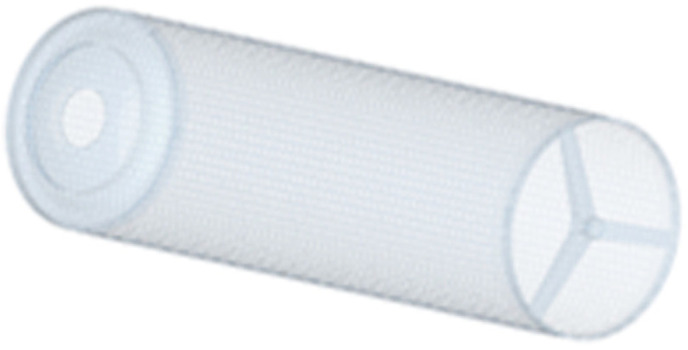
Simulation model of grading cylinder.

The geometric model of the cylinder was developed in Autodesk Inventor 2016. The completed model was exported as a STEP file and subsequently imported into EDEM 2018 software for simulation. The final cylinder model is presented in Fig 3.

### Stacking angle comparison test

The discrete element simulation parameters of maize seeds significantly influence the authenticity of subsequent simulation results. To validate the accuracy of these parameters, EDEM simulations of maize stacking angle were conducted. The experimental phase of this research involved physical measurements of stacking angles, with five repeated trials yielding a mean value of 27.18°. A corresponding DEM simulation model was developed, wherein parameter selection (see S1 Table) was guided by empirical measurements. Through Plackett–Burman screening, three statistically significant variables were identified: the dynamic friction coefficient of maize–maize, and the restitution coefficients of maize–maize and maize–steel plate. Subsequent steepest ascent experiments delineated optimal ranges for these key parameters. Non-significant factors were fixed at average values: the Poisson’s ratio of maize was 0.4, the density of maize was 1197 kg/m^3^, the shear modulus of maize was 1.37 × 10^8^ Pa, the static friction coefficient of maize–maize was 0.342, the static and dynamic friction coefficients of maize–steel plate were 0.379 and 0.048, respectively. A quadratic polynomial regression model of the stacking angle considering significant factors was obtained using the Box–Behnken test. Taking the actual stacking angle of 27.18° as the optimization objective, the optimal combination was formed: the dynamic friction coefficients of maize–maize was 0.054, and the restitution coefficients of maize–maize and maize–steel plate were 0.284 and 0.615, respectively. The stacking angle obtained from the simulation test is 28.25°. The relative error between the simulated and measured values was 3.94%, confirming the rationality of the selected simulation parameters.

### Interparticle contact forces

In Hertz-Mindlin model, the normal force component is based on Hertzian contact theory. The tangential force model is based on Mindlin-Deresiewicz work. Both normal and tangential forces have damping components where the damping coefficient is related to the coefficient of restitution. The tangential friction force follows the Coulomb law of friction model. The rolling friction is implemented as the contact independent directional constant torque model.

In particular, the normal force, *F*_n_ is a function of normal overlap *δ*n and is given by:


$$F_{n}=\frac{\text{4}}{\text{3}}E^{*}\sqrt{R^{*}}{\delta_{n}}^{\frac{\text{3}}{\text{2}}}$$ (1)

$$\frac{\text{1}}{E^{*}}=\frac{\left(1-vi^{2}\right)}{E_{i}}+\frac{\left(1-vj^{2}\right)}{E_{j}}$$ (2)

$$\frac{\text{1}}{R^{*}}=\frac{\text{1}}{R_{i}}+\frac{\text{1}}{R_{j}}$$ (3)

Where E^*^ is the equivalent Young’s Modulus, Pa;

R^*^ is the equivalent radius, mm.

With E_i_, ν_i_, R_i_, and E_j_, ν_j_, R_j_, being the Young’s Modulus, Poisson ratio and Radius of each sphere in contact. Additionally, there is a damping force, F_n_^d^, given by:


$$F_{n}^{d}=-2\sqrt{\frac{\text{5}}{\text{6}}}\beta \sqrt{S_{n}m^{*}}v_{n}\vec{rel}$$ (4)

$$m^{*}=(\frac{\text{1}}{m_{1}}+\frac{\text{1}}{m_{i}}{)}^{-1}$$ (5)

Where m^*^ is the equivalent mass, g;

$v_{n}^{\vec{rel}}$ is the normal component of the relative velocity, m/s;

β and S_n_ (the normal stiffness) are given by:


$$\beta =\frac{-\ln e}{\sqrt{\ln^{2}e+\pi^{2}}}$$ (6)

$$S_{n}=2E^{*}\sqrt{R^{*}\delta_{n}}$$ (7)

With e the coefficient of restitution. The tangential force, F_t_, depends on the tangential overlap δ_t_ and the tangential stiffness S_t_.


$$F_{t}=-S_{t}\delta_{t}$$ (8)

With


$$S_{t}=8G^{*}\sqrt{R^{*}\delta_{n}}$$ (9)

Here G^*^ is the equivalent shear modulus. Additionally, tangential damping is given by:


$$F_{t}^{d}=-2\sqrt{\frac{\text{5}}{\text{6}}}\beta \sqrt{S_{t}m^{*}}v_{t}^{\vec{rel}}$$ (10)

where $v_{t}^{\vec{rel}}$ is the relative tangential velocity. The tangential force is limited by Coulomb friction μ_s_F_n_ where μ_s_ is the coefficient of static friction.

For simulations in which rolling friction is important, this is accounted for by applying a torque to the contacting surfaces.


$$\tau_{i}=-\mu_{r}F_{n}R_{i}\omega_{i}$$ (11)

with μ_r_ the coefficient of rolling friction, R_i_ the distance of the contact point from the center of mass and ω_i_, the unit angular velocity vector of the object at the contact point.

### Simulation parameter settings

Based on sieving statistics, the mass proportions of large, medium, and small seeds were configured as 68%, 22%, and 10% respectively. The Hertz-Mindlin no-slip contact model was selected to simulate inter-seed collisions [13]. The time step was set to 8 × 10 ⁻ ⁶ s, the data saving interval to 0.01 s, and the simulation time to 2 s. Relevant studies [2,14] have shown that cylinder speed, inclination angle, and feeding rate are the primary factors influencing the performance of the cylindrical grader. To investigate seed separating law in the cylinder grading process, this study conducted simulation analyses using different values for these three key factors. The cylinder speed was set within the range of 14 r/min to 22 r/min, the inclination angle within the range of 0° to 4°, and the feeding rate within the range of 1,150 g/s to 1,350 g/s.

### Simulation results analysis

To ensure the robustness of the DEM simulation results, this study implemented several measures: Each parameter combination (rotational speed, inclination angle, and feeding rate) was independently simulated five times (n = 5) under identical initial conditions. The stability of key output parameters was evaluated using the coefficient of variation (CV), with acceptable thresholds set at CV ≤ 5% for axial velocity and CV ≤ 7% for the seed distribution uniformity index. Additionally, systematic grid convergence index (GCI) analysis and time step independence verification were conducted prior to finalizing the time step of 8 × 10 ⁻ ⁶ s. This comprehensive verification ensured solution stability across particle sizes, particularly for small particles.

### Axial mean velocity of maize seeds

#### Axial mean velocity under different cylinder speeds.

**Fig 4:**
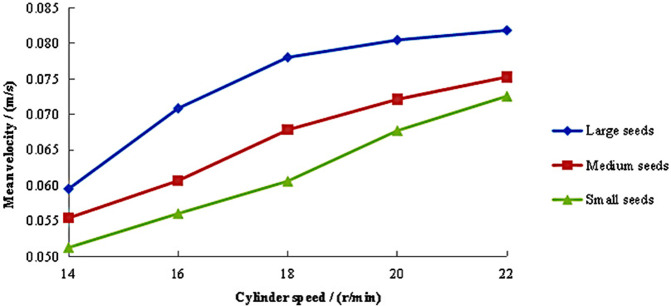
Axial mean velocity of seeds under different cylinder speeds.

The variation patterns of axial mean velocity for large, medium, and small maize seeds is illustrated in Fig 4, with different cylinder speeds (14–22 r/min) at a fixed inclination angle of 2° and feeding rate of 1,250 g/s. The results demonstrated that large seeds exhibited a progressive increase in axial velocity with higher cylinder speeds. A rapid velocity rise occurred within the 14–18 r/min range, followed by a decelerated growth rate at higher speeds. Notably, large seeds consistently maintained higher axial velocities than medium and small seeds across all tested speeds. Medium and small seeds showed approximately linear increases in axial velocity with rising cylinder speeds. Their growth trends were similar, though medium seeds persistently achieved slightly higher velocities than small seeds throughout the speed range.

#### Axial mean velocity under different inclination angles.

**Fig 5:**
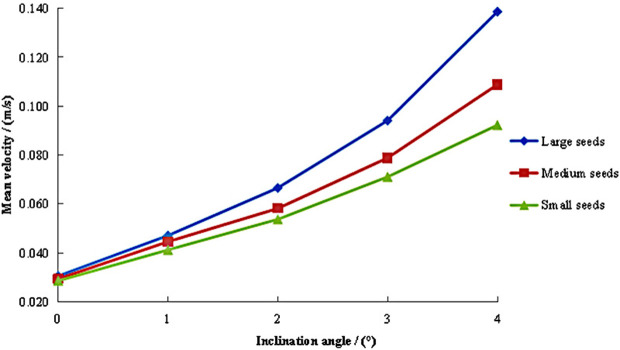
Axial mean velocity of seeds under different inclination angles.

The variation patterns of axial mean velocity for large, medium, and small maize seeds is illustrated in Fig 5, with different inclination angles (0°–4°) at a fixed feeding rate of 1,250 g/s and cylinder speed of 18 r/min. The results demonstrated that all seed sizes exhibited increased axial velocities with higher inclination angles. Overall, the axial mean velocity of large seeds consistently exceeded that of medium and small seeds, while also exhibiting the most pronounced variation with increasing inclination angles. This demonstrates that the axial mean velocity of large seeds is most significantly influenced by inclination angle. Within the 0°–1° inclination range, large seeds exhibited velocities comparable to medium and small seeds, with similar rates of change. However, when the inclination angle exceeded this range, the velocity variation of large seeds increased substantially. The medium and small seeds exhibited similar trends in mean velocity variation with inclination angles. When the inclination angle was less than 2°, their velocities remained comparable between seed sizes, and the variation amplitudes were relatively minor. When the inclination angle exceeded 2°, velocity increase amplitudes became significantly larger. Medium seeds consistently maintained higher velocities than small seeds throughout the angular range.

#### Axial mean velocity under different feeding rates.

**Fig 6:**
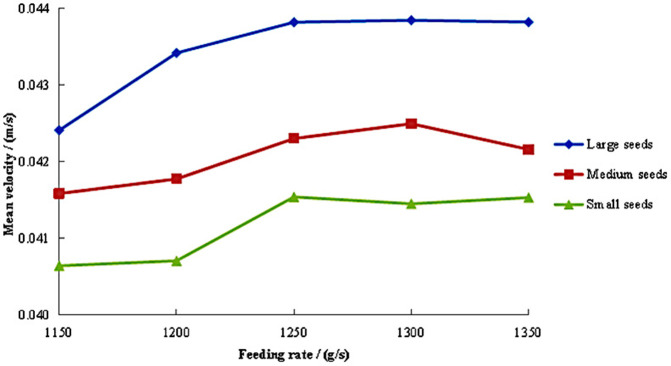
Axial mean velocity of seeds under different feeding rates.

The variation patterns of axial mean velocity for large, medium, and small maize seeds is illustrated in Fig 6, with different feeding rates (1,150–1,350 g/s) at a fixed inclination angle of 2° and cylinder speed of 18 r/min. The velocity trends exhibited distinct seed-size-dependent characteristics with increasing feeding rates. When the feeding rate was in the range of 1,150–1,200g/s, the velocity of large seeds increased rapidly. With the further increase of feeding rate, the velocity gradually remained stable, but the velocity of large seeds was significantly higher than that of medium and small seeds. The medium seeds exhibited a velocity profile characterized by an initial gradual increase followed by a slight decrease, demonstrating the smallest fluctuation range among all seed sizes. The velocity of small seeds showed a fluctuating upward trend while consistently maintaining the lowest values.

### Axial distribution of maize seeds

**Fig 7:**
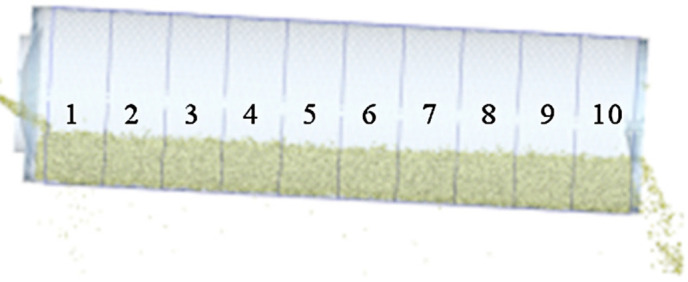
Seed axial distribution zoning.

Under the grading cylinder, 10 equally spaced zones were set along the seed discharge (axial) direction, as shown in Fig 7. Zone 1 was close to the inlet and zone 10 was close to the outlet. By statistically analyzing the seed quantity inside the cylinder and the sieved seeds in each zone, the seed distribution during the grading process was investigated. After the simulation started, the particle factory on the far left continuously produced maize seeds. Measurements of seed quantities in different zones began once the feed and discharge reached a steady state.

#### Axial distribution of maize seeds under different cylinder speeds.

**Fig 8:**
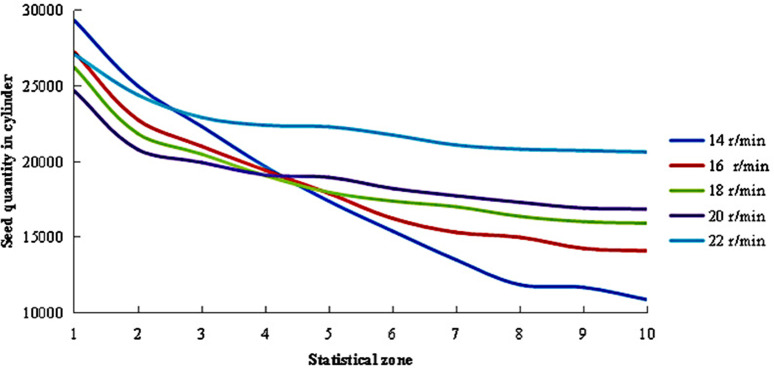
Distribution of seed quantities within the cylinder under different cylinder speeds.

When the cylinder operated at different rotation speeds, the distribution of seed quantity across different regions within the cylinder was observed, as shown in Fig 8. Since the newly generated seeds were initially deposited in Zone 1, this region consistently maintained the highest seed count. As the grading process progressed, the seeds gradually migrated toward the outlet on the right side. During this rightward movement, while the seed layer became progressively thinner, a portion of seeds smaller than the sieve apertures were sequentially separated. This resulted in a gradual decline in seed numbers from Zone 1 to Zone 10, stabilizing near Zone 8. Significant variations in the corresponding trends were observed with changes in cylinder speed. At 14 r/min, the variation trend in seed quantity manifested most significantly. As shown in Fig 4, the axial mean velocity of the seeds was lower at this speed, which increased the transit time from the inlet to the outlet. This extended the probability and duration of interaction between the seeds and the grading cylinder, thereby facilitating the sieving and separation of small and medium-sized seeds. With increasing cylinder speed, the trend of seed quantity reduction gradually slowed. At 22 r/min, the reduction in seeds was minimal, and the trend was the most gradual.

**Fig 9:**
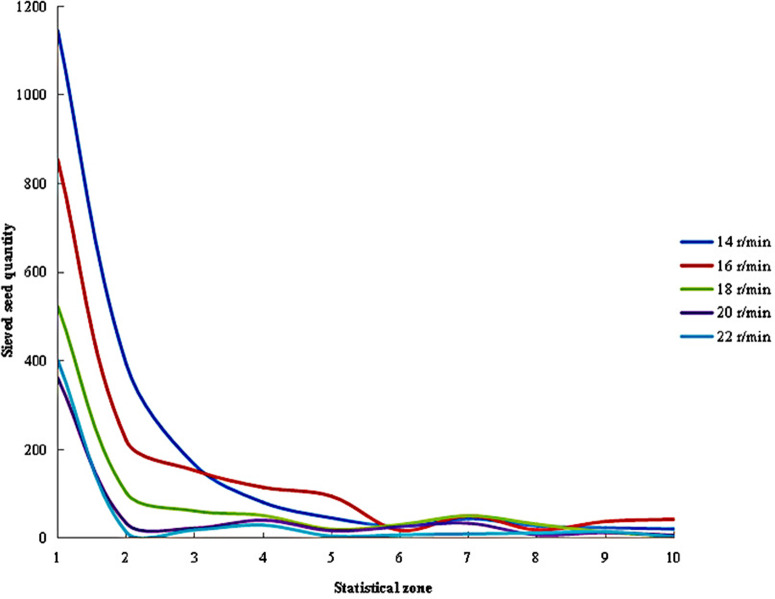
Distribution of sieved seed quantities under different cylinder speeds.

As shown in Fig 9, the distribution of sieved seed quantities across different regions varied with cylinder speeds. When the generated seeds initially fell into Zone 1, the absolute quantity and proportion of small and medium seeds were highest at this stage. Consequently, the number of sieved seeds in this zone remained the largest under all tested cylinder speeds, but it sharply decreased as the cylinder speed increased. As the grading process continued, the quantity and proportion of small and medium seeds gradually diminished from left to right within the cylinder. Correspondingly, the number of sieved seeds fluctuated and progressively declined, stabilizing near Zone 6. Overall, lower cylinder speeds resulted in more pronounced variation trends, whereas higher speeds led to flatter changes in seed quantity.

**Fig 10:**
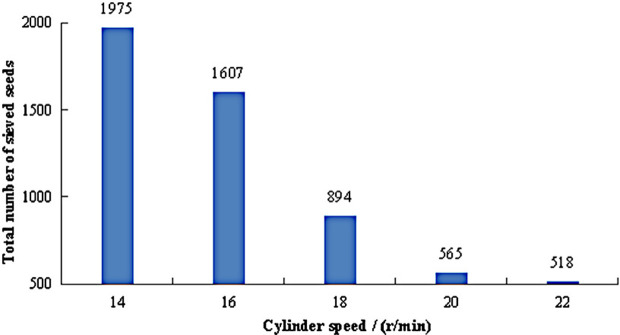
Total number of sieved seeds under different cylinder speeds.

The total number of sieved seeds under different cylinder speeds is shown in Fig 10. As the cylinder speed increased, the total number of sieved seeds gradually decreased. When the speed was set at 14 r/min, the axial velocity of the seeds was relatively low, allowing prolonged and sufficient contact between the seeds and the grading cylinder, resulting in a total of 1,975 sieved seeds. With further increases in speed, the axial velocity of the seeds rose, causing a reduction in the number of sieved seeds. Specifically, when the speed increased from 16 r/min to 18 r/min, the total number of sieved seeds dropped by approximately 44%. At 22 r/min, the total number of sieved seeds was only 518, indicating that a significant portion of small and medium seeds failed to be separated, severely compromising the grading qualification rate. This phenomenon occurred because as the cylinder speed increased, the axial mean velocity of the seeds also rose, reducing both the probability and duration of contact between the seeds and the cylinder, thereby decreasing the likelihood of sieving. Additionally, higher cylinder speeds intensified collisions between the seeds and the cylinder, hindering their relative slip movement and further complicating the sieving process. Consequently, maintaining the cylinder speed within the range of 14–18 r/min was conducive to improving the grading qualification rate.

#### Axial distribution of maize seeds under different inclination angles.

**Fig 11:**
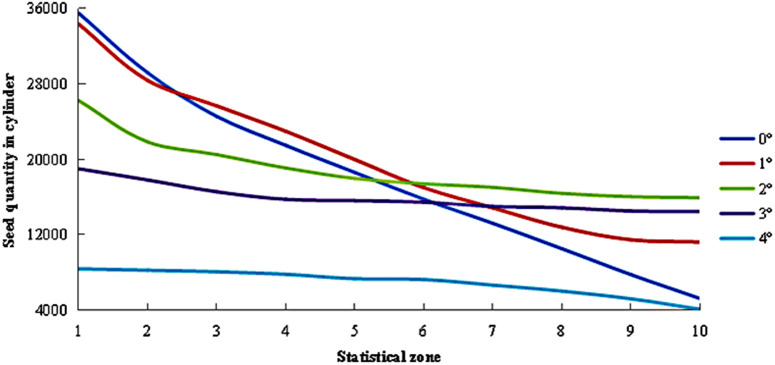
Distribution of seed quantities within the cylinder under different inclination angles.

As shown in Fig 11, the distribution of seed quantities across different regions within the cylinder varied with inclination angles. Under all tested angles, Zone 1 consistently exhibited the highest seed count, with seed numbers gradually decreasing from Zone 1 to Zone 10. When the inclination angle was set at 0°, the seed quantity within the cylinder showed a pronounced and continuous decline, with significant variations persisting even near Zone 10. This observation indicated that at 0°, the seeds’ axial velocity was low, allowing prolonged and thorough contact with the cylinder. This condition facilitated the effective and reliable separation of small and medium seeds, thereby enhancing grading quality. As the inclination angle increased beyond 1°, the seed count in Zone 1 sharply decreased. Simultaneously, the overall variation trend in seed distribution gradually flattened. This occurred because higher inclination angles significantly accelerated the seeds’ axial velocity, causing seeds in Zone 1 to rapidly migrate toward Zone 10, resulting in more uniform seed distribution across regions. The greater the axial velocity, the fewer seeds were sieved, and the more similar the seed quantities became across all zones.

**Fig 12:**
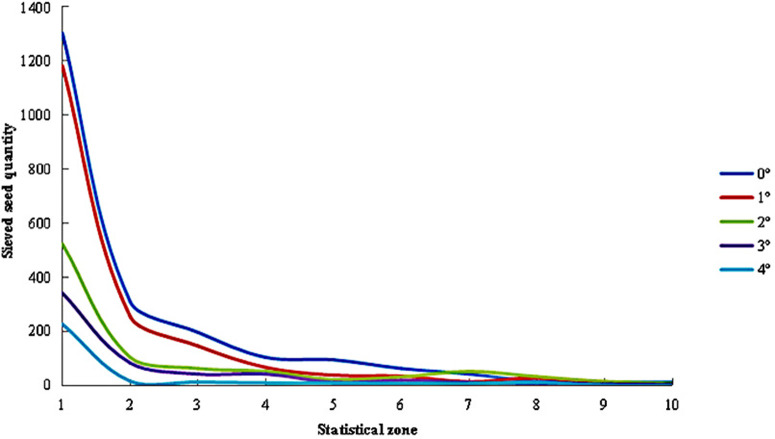
Distribution of sieved seed quantities under different inclination angles.

The distribution of sieved seeds across different regions under varying inclination angles is presented in Fig 12. When the inclination angle was set at 0° and 1°, the trends and numerical values of sieved seed counts across regions were highly similar. At this point, the number of sieved seeds in Zone 1 remained around 1,200, but it sharply decreased to approximately 300 by Zone 2. From then on, the reduction in seed counts across subsequent regions became more gradual, stabilizing after Zone 7. However, when the inclination angle increased to 2°–4°, the number of sieved seeds in Zone 1 dropped significantly compared to lower angles. Subsequently, seed counts across other regions showed minimal variation, with only a slow decline observed after Zone 3.

**Fig 13:**
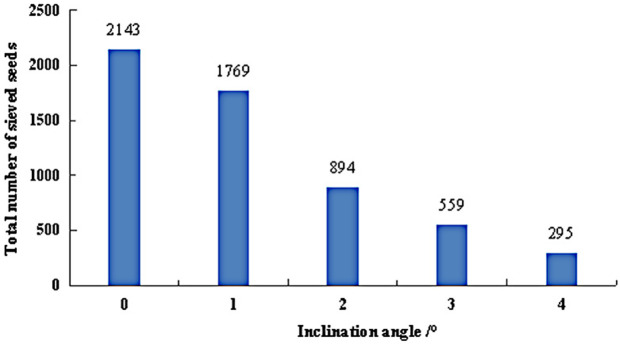
Total number of sieved seeds under different inclination angles.

The total number of sieved seeds under varying inclination angles is presented in Fig 13. The results indicated that as the inclination angle increased, the total count of sieved seeds progressively decreased. At 0°, the seeds’ axial velocity remained low, facilitating the reliable separation of small and medium seeds. Consequently, the total number of sieved seeds reached its peak at 2,143. However, as the inclination angle gradually increased, the seed velocity rose significantly, reducing the contact time between seeds and the grading cylinder. This insufficient interaction hindered the thorough separation of small and medium seeds, leading to a steady decline in the total sieved count. When the inclination angle increased from 1° to 2°, the seed velocity surged, resulting in a sharp 50% reduction in the total number of sieved seeds. At 4°, the total sieved count dropped to its minimum of 295, which was merely 13.8% of the count recorded at 0°. This significant decrease would severely compromise the quality of the grading process. Therefore, maintaining the cylinder inclination angle within the 0°–2° range was optimal for enhancing the grading qualification rate.

#### Axial distribution of maize seeds under different feeding rates.

**Fig 14:**
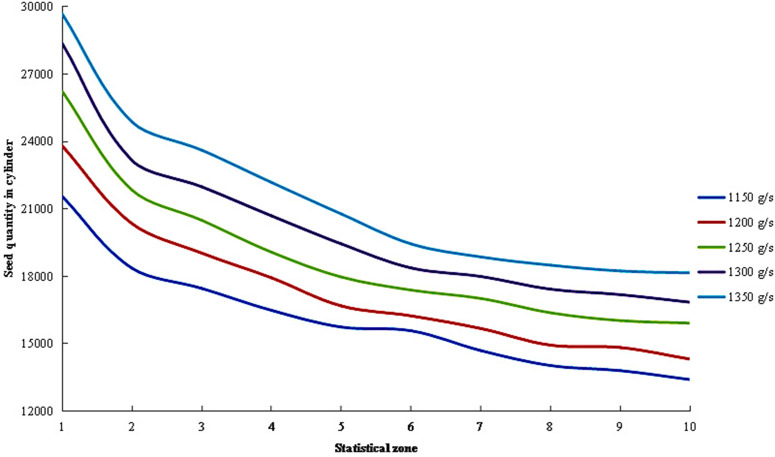
Distribution of seed quantities within the cylinder under different feeding rates.

The distribution of seed quantities across different regions within the cylinder under varying feeding rates is presented in Fig 14. The results showed that the lower the feeding rate, the fewer the seeds in each region of the cylinder, whereas the higher the feeding rate, the greater the seed count in the corresponding regions, with a consistent trend observed across all rates. This occurred because the cylinder speed and inclination angle remained constant, ensuring uniform seed processing capacity. As the feeding rate increased, the number of seeds entering the cylinder per unit time rose, leading to a gradual increase in seed quantities across all regions.

**Fig 15:**
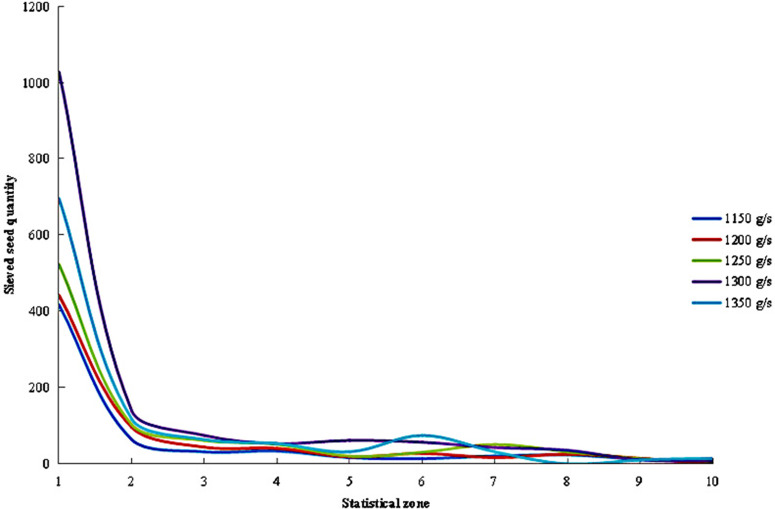
Distribution of sieved seed quantities under different feeding rates.

The distribution of sieved seeds across different regions under varying feeding rates is shown in Fig 15. The results indicated that when the feeding rate gradually increased from 1,150 g/s to 1,250 g/s, the number of sieved seeds in each region showed a slow upward trend, with minimal differences observed. In this range, the highest count in Zone 1 reached approximately 500, followed by a sharp decline to around 100 in Zone 2, after which the numbers decreased gradually. When the feeding rate was raised to 1,300 g/s, the number of sieved seeds in Zone 1 surged to over 1,000. This was followed by a sharp drop in Zone 2, after which the counts decreased slowly from Zone 4 onward. Further increasing the feeding rate to 1,350 g/s caused the seed count in Zone 1 to drop to about 700.

**Fig 16:**
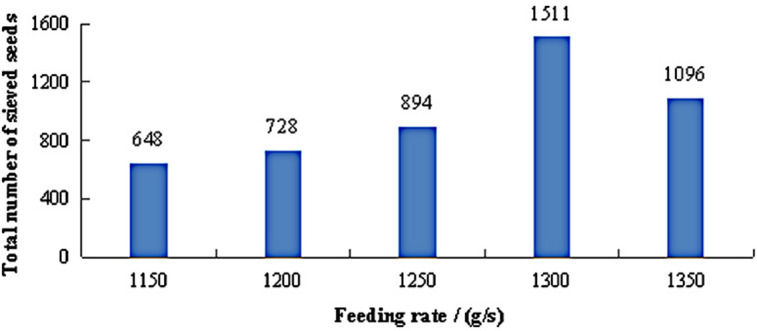
Total number of sieved seeds under different feeding rates.

The total number of sieved seeds under different feeding rates is shown in Fig 16. As the feeding rate increased from 1,150 g/s to 1,300 g/s, the total number of sieved seeds gradually rose. At a feeding rate of 1,300 g/s, the highest number of sieved seeds was recorded, reaching 1,511. However, when the feeding rate was further increased to 1,350 g/s, the number of sieved seeds dropped to 1,096. This phenomenon can be explained by the constant processing capacity of the cylindrical grader when maintaining fixed operational parameters (rotation speed: 18 r/min; inclination angle: 2°). Experimental evidence suggests an optimal processing capacity of approximately 1,300 g/s. Below this critical value, enhanced feeding rates increased both the total seed input and the absolute quantity of medium/small seeds available for separation, thereby improving sieving efficiency. When exceeding 1,300 g/s, seed accumulation within the cylinder occurred due to input-output imbalance, which impaired the tumbling action and limited seed-wall contact opportunities. Consequently, the optimal feeding rate range of 1,250−1,350 g/s was established to maximize classification efficiency while maintaining operational stability.

### Experiment

Through the simulation analysis of maize seed cylindrical grading, the optimal range of parameters was obtained. Using EDEM2018 simulation software, an orthogonal experiment was conducted to determine the better parameter combination. Comparing the simulation test results under the optimal parameter combination with the physical test results verified the rationality of the maize seed cylindrical grading simulation analysis.

### Orthogonal experiment

#### Orthogonal experiment design.

Relevant study [7] indicate that the qualification rate and productivity are crucial operational indicators for cylindrical graders, while cylinder speed, inclination angle, and feeding constitute key factors affecting grading performance. Concurrently, simulation experiments demonstrate that when these three critical parameters are maintained within the ranges of 14–18 r/min, 0°–2°, and 1,250–1,350 g/s respectively, seed distribution within the cylinder achieves relative uniformity, thereby facilitating effective sieving. A three-factor, three-level experimental design was established according to the L9 (3^4^) orthogonal array, comprising 9 test groups. The corresponding factors and their levels are presented in S3 Table.

#### Experimental results and analysis.

The orthogonal test results for maize seed cylindrical grading were obtained according to the factors and levels designed in S3 Table, as detailed in S4 Table. To analyze the impact of different experimental factors on grading performance indicators, a range analysis was conducted on the test results, with the outcomes summarized in S5 Table.

The results of the range analysis showed that the importance of different factors affecting the qualification rate was ranked as *A* > *B* > *C*, and the optimal combination was *A*_2_*B*_1_*C*_1_. For productivity, the ranking was *B* > *C* > *A*, with the optimal combination being *A*_3_*B*_3_*C*_2_. For maize seeds, since the subsequent precision sowing had high requirements for seed size, it was necessary to prioritize maintaining a high qualification rate, while appropriately relaxing the demands on productivity. Through comprehensive comparison and analysis, the best overall performance was achieved with the combination *A*_2_*B*_1_*C*_2_, corresponding to cylinder speed of 16 r/min, inclination angle of 0°, and feeding rate of 1,300 g/s. This combination was Test No. 4 in S4 Table. Simulation tests were repeated five times, and the results are presented in S6 Table.

To verify the rationality of the simulation analysis, maize seed cylindrical grading tests were conducted at the Nanjing Institute of Agricultural Mechanization. Before the tests, the seeds underwent wind screening to remove impurities. During the trials, after the grader operated stably, both the qualification rate and productivity were measured separately. The calculation formulas for the qualification rate and productivity are presented in Equations (12) and (13), respectively [15].


$$R=\frac{M_{\text{1}}}{M}\times 100\%$$ (12)

where *R* is the qualification rate, %;

*M*_1_ is the mass of qualified grains in the sample measured at the main outlet, g;

*M* is the total mass of sample measured at the main outlet, g.


$$P=\frac{W}{T}$$ (13)

where *P* is the productivity, g/s;

*W* is the total sample mass measured at each outlet, g;

*T* is the measurement time, s.

The optimized combination was used to conduct five repeated trials [16–18], and the results are presented in S7 Table. The comparison of simulation and validation test results are presented in S8 Table. The results showed that the relative errors between the validation test results of maize seed cylinder grading and the simulation results were all below 5%, thereby confirming the rationality of the simulation analysis for maize seed cylinder grading.

## Discussion

### Simplification of maize seed model

In this study, the maize seed was simplified as a model composed of four spheres to reduce computational load and improve simulation efficiency. Model simplification is a common method because no matter how many spheres are used in the seed model, it cannot fully replicate the actual shape of the seed. To ensure the simulation closely reflects real-world conditions, this study calibrated the model parameters of maize seeds by comparing the simulated and measured values of seed stacking angle, as well as the performance indicators between simulated seed grading tests and physical experiments. The results show that the relative errors between simulated and measured values are all less than 5%, indicating that the experimental results are insensitive to model simplification. The simplified model has minimal impact on grading accuracy.

### Error source analysis

The relative errors between simulated and measured values stem from: 1. Dynamic friction variations: Prolonged grading operations induce surface wear and dust adhesion on maize seeds due to repeated inter-seed and seed-wall collisions, dynamically altering friction coefficients—a phenomenon unaccounted for in EDEM static friction modeling. 2. Feeding instability: Physical tests exhibited intermittent pulse disturbances from feeder limitations, compromising operational consistency. As shown in S6–S8 Tables, when the feeding rate was set to 1300 g/s, the simulation experiments yielded standard deviations of 0.91 for the qualification rate and 14 for productivity. In contrast, the validation experiments showed higher standard deviations of 2.05 and 35 for these metrics, respectively. The results indicate that physical experiments exhibited greater fluctuations in all performance indicators.

### Influence of parameters on operational quality

Multiple studies [7,19,20] have consistently identified cylinder speed, inclination angle, and feeding rate as the critical parameters affecting the performance of cylindrical grader. However, discrepancies in material properties (e.g., particle size, shape, density, and friction characteristics) across different graded materials lead to pronounced variations in motion dynamics and distribution patterns during the grading process. Consequently, the priority ranking of these critical parameters varies significantly. Furthermore, the operational ranges investigated for these parameters differ substantially across studies, resulting in divergent magnitudes of their effects on grading quality. Therefore, the findings of this study have certain limitations and are only applicable to maize seeds, with no generalizability to other seed types.

### Recommendations for future research

Based on findings from both simulation and physical experiments, it is evident that non-uniform seed distribution within the cylindrical grader limits its grading efficiency. Seed accumulation near the feeding inlet (cylinder’s proximal section) overwhelms the grading capacity, leading to incomplete separation of overloaded particles. Sparse seed distribution near the discharge outlet (distal section) results in idle grading area. This phenomenon arose because a smaller inclination angle yielded higher qualification rates. However, at low angles, reduced axial velocity hindered seed movement from the front to the rear. To address this, future studies could incorporate deflector plates or spiral devices to enhance seed distribution uniformity and optimize grading efficiency. Due to equipment and time constraints, this study did not incorporate mixing components such as spiral devices, and related experiments have not yet been conducted. This represents the other limitation of the present study. However, existing research [2] has shown that spiral devices can effectively improve material distribution uniformity and enhance the performance of rotary screens.

## Conclusion

The discrete element model of maize seed cylindrical grading was simulated under varying cylinder speeds (14–22 r/min), inclination angles (0°–4°), and feeding rates (1,150–1,350 g/s). Orthogonal experiments and range analysis were conducted to determine the importance order of each factor on qualification rate: cylinder speed, cylinder angle, and feeding rate. For productivity, the importance order was: inclination angle, feeding rate, and cylinder speed. The optimal parameter combination for achieving high-quality grading was: cylinder speed of 16 r/min, inclination angle of 0°, and feeding rate of 1,300 g/s. At this point, the qualification rate was 93.21%, and the productivity was 1,033 g/s. A validation experiment yielded a qualification rate of 90.15% and a productivity of 985 g/s. The relative errors between simulated and measured values were less than 5%, validating the rationality of the maize seed simulation analysis.

## Supporting information

S1 Table Parameter of maize seed of DEM. The relevant model parameters obtained by referring to relevant literature.
(XLSX)

S2 Table Parameter of grading cylinder of DEM. The parameters of the grading cylinder were obtained from the materials manual.
(XLSX)

S3 Table Orthogonal experimental factors and levels. The table lists three levels of parameter setting for the three factors in the test.
(XLSX)

S4 Table Results of orthogonal test. (XLSX)

S5 Table Range analysis results. (XLSX)

S6 Table Simulation test results. (XLSX)

S7 Table Validation test results. (XLSX)

S8 Table Comparison of simulation and validation test results. (XLSX)
